# Improvement in feed efficiency and reduction in nutrient loading from rainbow trout farms: the role of selective breeding

**DOI:** 10.1093/jas/skac214

**Published:** 2022-06-09

**Authors:** Antti Kause, Antti Nousiainen, Heikki Koskinen

**Affiliations:** Natural Resources Institute Finland (Luke), Animal Genetics, Jokioinen FI-31600, Finland; Natural Resources Institute Finland (Luke), Aquaculture Solutions, Kuopio FI-70210, Finland; Natural Resources Institute Finland (Luke), Aquaculture Solutions, Kuopio FI-70210, Finland

**Keywords:** aquaculture, breeding program, feed intake, feed conversion ratio, genetic trend

## Abstract

Resource efficiency, the ratio of inputs to outputs, is essential for both the economic and environmental performance of any sector of food production. This study quantified the advancement in the feed conversion ratio (FCR) and reduction in nutrient loading from rainbow trout farming in Finland and the degree to which genetic improvements made by a national breeding program have contributed to this advancement. The study combined two datasets. One included annual records on farm-level performance of commercial rainbow trout farms from 1980 onwards, and the other included individuals across eight generations of the national breeding program. The data from the commercial farms showed that from 1980 onwards, the farm-level feed conversion ratio improved by 53.4%, and the specific nitrogen and phosphorus loading from the farms decreased by over 70%. Hence, to produce 1 kg of fish today, only half of the feed is needed compared to the 1980s. The first generation of the breeding program was established in 1992. The FCR was not directly selected for, and hence, the genetic improvement in the FCR is a correlated genetic change in response to the selection for growth and body composition. Since 1992, the estimated genetic improvement in the FCR has been 1.74% per generation, resulting in a cumulative genetic improvement of 11.6% in eight generations. Genetic improvement in the FCR is estimated to be 32.6% of the total improvement in the FCR observed at farms, implying that genetic improvement is a significant contributor to resource efficiency. The use of genetically improved rainbow trout, instead of the base population of fish, reduces feed costs by 18.3% and total production costs by 7.8% at commercial farms (by −0.266€ per kg of ungutted fish). For phosphorus and nitrogen, it can be assumed that the use of fish material with an improved FCR also leads to 18.3% less nitrogen and phosphorus flowing into an aquatic environment. Such improvements in resource efficiency are win–wins for both industry and the environment—the same amount of seafood can be produced with significantly reduced amounts of raw materials and reduced environmental impact.

## Teaser

This study shows that the feed conversion ratio of farmed rainbow trout has been improved by selective breeding, and hence, the same amount of seafood can be produced with significantly reduced amounts of raw materials and reduced environmental impact.

## Introduction

During the last decade, aquaculture has expanded globally at an annual rate of 5%–6%. In 2016, approximately 47% of consumed fish originated from aquaculture, and aquaculture production exceeded the production of beef (FAO, 2018). Resource efficiency, the ratio of inputs to outputs, is essential for profitability and the environmental impact of the food industry. In this study, we quantified the trend of the improvement in resource efficiency in rainbow trout farming in Finland over four decades and the degree to which animal breeding contributed to this improvement.

The feed conversion ratio (FCR) is the ratio of feed intake to fish biomass growth. In animal production, the FCR is a major determinant of the profitability of the industry and environmental sustainability. First, in aquaculture, feed cost is the largest single cost category in primary production. For example, in rainbow trout farming, the feed costs are approximately 43% of the total production costs (Kankainen et al., 2016a, b). Second, fish farming in net pens and raceways has low energy needs and produces limited amounts of CO_2_ emissions, whereas the production of fish feed is energy intensive and accounts for a large share of CO_2_ emissions from aquaculture (Ziegler et al., 2013). Third, nutrient loading to the environment is determined by the amount of feed provided and its composition but also by the retention efficiency with which the fish convert feed and its nutrients into tissue growth. In fact, farmed fish such as Atlantic salmon retain only 45% of the ingested protein (nitrogen) (Halver and Hardy, 2002). In these three cases, an improved FCR reduces both feed costs and the environmental footprint.

Nitrogen (N) and phosphorus (P) are the main nutrients that micro- and macroalgae need for growth. Elevated levels of these nutrients in aquatic environments cause eutrophication. Specific nutrient loading from aquaculture can be quantified as the amount of nutrients ending up in water (kg) per 1000 kg of fish produced. Similar to the FCR, environmental loading defined in this way is a measure of the efficiency of aquaculture production. Human-induced eutrophication is regarded as a major negative impact in many instances, including environments in which aquaculture is practiced in Nordic countries.

In Finland, a national selective breeding program for rainbow trout was started in 1989 (Siitonen, 1986; Kause et al., 2005). The FCR itself is not recorded and directly selected for in any fish breeding programme. However, in some species, such as rainbow trout, the FCR is modestly genetically correlated with growth rate, lipid deposition, and lean growth, a phenomenon that is also prevalent in other farm animals, such as chickens and pigs (Quinton et al., 2007; Quillet et al., 2007; Kause et al., 2016; Knap and Kause, 2018). This allows breeders to improve the FCR indirectly by selecting for these correlated traits, as is practiced in the Finnish breeding program for rainbow trout (Kause et al., 2005, 2007b, 2016; Knap and Kause, 2018). In addition, farm management, feeding, and feed formulation have all improved during recent decades.

In this study, the objectives were 1) to quantify the long-term trend from 1980 onwards in the farm-level FCR and in specific N and P loading in the commercial rainbow trout industry in Finland; 2) to quantify the degree to which selective breeding has genetically improved the FCR and the consequent reduction in nutrient loading; and 3) to calculate the economic benefit of using modern genetically improved rainbow trout compared to using rainbow trout dating back to the early 1990s.

## Material and Methods

### Ethical approval

This study was conducted using two datasets. One dataset included annual records on farm-level performance at commercial rainbow trout farms, and the other included records on individual fish of the national breeding program.

The data on commercial farms were extracted from an existing database that forms the basis for regulators to evaluate whether farms operate according to their farming licenses. The study of the breeding program was conducted according to the guidelines established by the Natural Resources Institute Finland (Luke). The study was performed in accordance with Finnish animal welfare legislation and complied with the directive 2010/63/EU implemented in Finnish legislation in the Act on the Use of Animals for Experimental Purposes (62/2006). All experimental fish were anaesthetized with tricaine methanesulfonate before sampling to minimize suffering.

### Commercial farm data

To assess the phenotypic change across decades in the FCR and nutrient loading, data from commercial rainbow trout farms were collected. Information on the annual values of the amount of feed provided, fish biomass grown, and specific phosphorus and nitrogen loading at commercial fish farms was extracted from the database of the Centre for Economic Development, Transport and the Environment (ELY Centre, Finland). The ELY Centre is a governmental authority monitoring the licensing of fish farms. Each farm submits the abovementioned information annually to the ELY Centre. The data consist of all the farms that have been approved of a farming license in the coastal areas of mainland Finland between 1980 and 2016. In 1980, the number of farms was 12, and the number of farms rose rapidly to 108 farms by 1985. The maximum was 381 farms in 1991, and in 2016, there were 159 farms.

For each farm in each year, the farm-level feed conversion ratio (FCR_Farm_) was calculated as


$$\begin{array}{ll} FCR_{Farm}\left(kg\;feed/kg\;f\;ish\right)= & \;Total\;feed\;use\left(kg\right)\\ & /Fish\;biomass\;growth\left(kg\right). \end{array}$$

It is important to note that the FCR_Farm_ is not the same as the FCR recorded on an individual fish (FCR_Ind_). For the FCR_Farm_, the amount of biomass is influenced by both growth and survival. If there is a mismatch between the amount of feed provided and fish biomass, for instance, because recent fish mortality and feeding are not adjusted for it, the consequent overfeeding will deteriorate the FCR_Farm_.

For each farm in each year, nutrient loading was calculated as


$$\begin{array}{ll} FCR_{Farm}\left(kg\;feed/kg\;f\;ish\right)= & \;Total\;feed\;use\left(kg\right)\\ & /Fish\;biomass\;growth\left(kg\right). \end{array}$$

It is important to note that the FCR_Farm_ is not the same as the FCR recorded on an individual fish (FCR_Ind_). For the FCR_Farm_, the amount of biomass is influenced by both growth and survival. If there is a mismatch between the amount of feed provided and fish biomass, for instance, because recent fish mortality and feeding are not adjusted for it, the consequent overfeeding will deteriorate the FCR_Farm_.

Specific nitrogen loading was calculated in the same manner, but P was substituted by N. The P and N concentrations of rainbow trout were 0.4% and 2.75%, respectively. Farmers record the concentrations of P and N in feeds from the certificates provided by the feed manufacturers. To provide some background, in 1999, the percentages of phosphorus and nitrogen in feeds were typically 0.91% and 6.8%, respectively, with a downward trend over time (Wideskog, 2000; Seppälä et al., 2001). It should be noted that the amount of feed sold is 14% higher than the records of feed provided, and hence, it is possible that the FCR_Farm_ value is in reality slightly higher than reported (Wideskog, 2000).

### Data from the rainbow trout breeding program

The data from the Finnish national breeding programme maintained by the Natural Resources Institute Finland (Luke) were used to estimate the genetic trend in the FCR across decades. The data had a total of 23 year classes from 1992 to 2015 in which fish traits have been recorded on pedigreed individuals through a 3- to 4-yr life cycle from juvenile to adult fish. This provided 8 generations and 7 episodes of genetic selection, plus the base population of the 1989 and 1990 year classes. The breeding program has been maintained at Luke’s Tervo aquaculture station in central Finland.

The data consisted of a total of 547,246 individuals with 537,262 individuals with phenotypes and an additional 9,984 individuals with only pedigree information. It should be noted that the highest sample size was for tagging weight, Weight_1_, whereas the other traits had much lower sample sizes (Table 1). The data included 3260 sires, 3270 dams, and 5821 full-sib families. The sires were mated to an average of 1.8 dams, and the dams were mated to an average of 1.8 sires. Each class had 109–341 families. During the grow-out period for each class, fish were either kept at the freshwater nucleus station or sent to one or two sea test stations (Table 1).

**Table 1.** Sample size (*n*), trait means, phenotypic variance (*V*_P_), and year classes in which the traits of the breeding program were recorded for (A) breeding values estimation and for (B) estimation of genetic parameters for specific traits in year classes 1992–2015

	*n*	*Mean*	*V*_*P*_	Year classes with or without records
A. Traits in breeding value evaluation				
Weight_1_, g	525 247	61.2	482.4^1^	Only 2010 missing
Weight_2_, g	94 206	1021	38 085^1^	2008,2009,2011,2012,2015 missing
Weight_3_, g	95 309	2178	143 409^1^	2012 missing
Sea weight_2_, g	82 168	1162	84 779^1^	1993,1995,2010 missing
Sea gutted weight_2_, g	74 824	1028	65 039^1^	1992, 1993,1995,2002,2010 missing
Sea visceral%, %	78 829	11.9	2.7723^1^	1992, 1993,1995,2002,2010 missing
Sea survival_2_, proportion	93 614	0.678	0.20006^1^	1992,1993,1995,1996,1997, 1998,1999,2010 missing
B. Traits in separate experiments to estimate genetic parameters				
Sea FCR_Ind_, g feed/ g weight gain	692	1.26	0.3246^2^	Recorded in 2001
Sea fillet%, %	2 671	64.75	6.3064^3^	Recorded in 2003, 2004
Sea muscle lipid%_BW_, %	998	7.70	4.384^4^	Recorded in 2001

Estimated using the models given in Table 2.

Trait ‘LifeFCR_Indicator_’ of Kause et al. (2016). Original mean = 0.845E-02 and *V*_P_ = 1.46E-05, rescaled here to reflect the FCR_Farm_ of year 2002 in the commercial farm data.

Trait ‘Fillet percentage’ of Kause et al. (2007b).

Trait ‘Muscle lipid%_[BW]_’ of Kause et al. (2016).

The parents for each generation were selected based on their estimated breeding values (EBVs) for growth (since 1992), age at maturity (since 2001), external appearance (since 2001), skeletal deformations (since 2002), fillet color (2003–2012), cataracts caused by *Diplostomum* parasites (since 2003), percentage of viscera weight out of body weight (2005) and survival (since 2010). The practice of estimating EBVs is the same as described in the section “Genetic trend analysis.” To control the rate of inbreeding, the optimal genetic contribution method has been used since 2002 to select the individuals with the highest selection index values who are not too closely related and to assign the number of matings and mating pairs of the selected individuals (Kause et al., 2005). Each year, parental fish are mated at the Tervo freshwater nucleus station during April–June.

The year class 2001 was the first one in which the breeding program had two selection lines, one for maintaining a steady age at maturity (Growth-line) and a new selection line in which one of the main breeding goals was delayed age at maturity (Delayed maturity-line) (Ritola et al., 2006). EBVs were estimated simultaneously for both lines with the full data presented here. In the current study, however, genetic trends were presented only for the Growth-line. Only this line was used throughout the years analyzed here, and it is by far the more commonly used line at commercial farms (>70% of the material sold from the breeding program).

After mating, full-sib egg batches were incubated separately within subdivided trays. At the eyed-egg stage, each full-sib family was transferred to one of two 150-liter indoor tanks. Eggs hatched in July, and the first feeding occurred in August. After 2 to 3 wk of growth in the tanks (at a body weight of 2 g), full-sib families were equalized to similar family size of 150 individuals. Thereafter, the full-sib families were kept separately in 150-liter indoor tanks until the start of individual tagging in November.

All the fingerlings were individually weighed to the nearest 0.1 g during tagging (Weight_1_) when they had grown for one growing season in freshwater family tanks. At tagging, each family was split into two groups: one group was held in open raceways at the nucleus as breeding candidates, and the other group was transferred into net pens at one or two commercial sea stations located in the Baltic Sea. Over 80% of commercial production occurs at sea.

From 2000 onwards, within-family selection for Weight_1_ has been practiced to maximize genetic gain in the nucleus (Martinez et al., 2006). Within-family selection was practiced during tagging by leaving the largest fish within a family in the nucleus and transferring the second largest fish to the two sea stations. The remaining untagged fish within a family were weighed as a group (*w*), counted (*n*), and culled. Then, for each family, it was assumed that Weight_1_ had a normal distribution with a mean value calculated from the data. The standard deviation of a family (SD) was calculated based on only the unculled observations above the mean (for 1/2 the SD) and assuming a symmetrical normal distribution (to obtain the full SD). Individual Weight_1_ records for the culled individuals were then randomly drawn from the left-hand side of the normal distribution, below the body weight threshold that was used for culling, while simultaneously maintaining the original mean Weight_1_ of the culled group. These generated individual records were added to the data. Simulations have shown that this practice prevents selection bias, i.e., restores trait variances to the level before within-family selection (Janhunen et al., 2014). On average, 46 individuals from each family were individually weighed and tagged (typically 15 were sent to sea and 25 held at the nucleus), whereas data were generated for an average of 60 untagged fish per family.

At the freshwater nucleus, after the second growing season, fish were weighed to the nearest 1 g during April–June (Weight_2_). Fish were classified according to the presence or absence of deformed skeletal structures through visual inspection (Kause et al., 2005, 2007a). Deformations were recorded based on the external characteristics of the live fish through visual inspection Deformed here refers to fish with deformed skeletal structures of the head, neck, back, or tail. A fish with any form of deformation received a score of one, and a normal fish received a score of zero. Because the recording was based on external characteristics, the average incidences given are underestimates of the true deformity rates. The lenses of the fishes’ eyes were evaluated for cataracts caused by parasitic *Diplostomum* spp. eye flukes (Kuukka-Anttila et al., 2010). A visual eye examination was carried out on the fish by a trained person, and cataracts were scored as 0 = healthy eyes, 1 = one opaque eye, and 2 = both eyes opaque. Furthermore, sex-specific maturity traits were recorded (11 = maturing male, 12 = maturing female, 10 = immature male, 20 = immature female, 9 = sex or maturity unknown). At the freshwater nucleus, fish were classified by sex and maturity after both the second (based on visible external characteristics) and third growing seasons (visually and using an ultrasound device) (Kause et al., 2003). The fish maturing at the age of 2 yr were removed from the population. Fish whose sex could not be determined or who died were coded as sex not known. However, after the third growing season, live fish of unknown sex were late-maturing individuals whose gonads were not visible at this stage, and they were coded as immature females. Females typically mature one year later than males, and each fish matures only once during its lifetime. The 3-yr-old fish were weighed in late September–November (Weight_3_) (Table 1).

At the sea stations, after one freshwater growing season (fingerling period) and one sea growing season, fish were weighed to the nearest 1 g during October–April (Sea weight_2_), and their grow-out survival between tagging and the end of the grow-out period at sea (Sea survival_2_) was recorded. Male fish were classified as mature and immature. At sea, fish were harvested before female maturation, and hence all females at sea were immature. At the same time, gutted body weight was recorded to the nearest 1 g (Sea gutted weight_2_) to calculate the percentage of entrails (Sea visceral% = 100 (Sea weight_2_ − Sea gutted weight_2_) / Sea weight_2_) (Table 1).

To complete the genetic trend analysis, the phenotypic and genetic parameters estimated in our previous studies of the traits Sea FCR_Ind_, Sea muscle lipid%_BW_ (Kause et al., 2016) and Sea fillet% (Kause, 2007b) were also used in the present study (Table 1). To record the trait Sea FCR_Ind_, feed intake was recorded on individuals using the X-ray technique (Kause et al., 2016), and FCR_Ind_ was calculated as follows: Feed intake/Body weight gain. Feed intake was recorded 9 times for fish growing from an initial average weight of 143.5 g to the final average weight of 2113 g. The trait Sea muscle lipid%_BW_ was chemically determined lipid% of a piece of a cross-sectional slice of a fillet, statistically corrected for by using final weight as a fixed regression term (at an average weight of 2113 g). Sea FCR_Ind_ and Sea muscle lipid%_BW_ were recorded at the freshwater nucleus (Kause et al., 2016), but their genetic parameters were assumed to be applicable to a sea environment. Sea fillet% was recorded on the routine breeding programme fish reared at sea, and the trait was calculated as the percentage of the weights of untrimmed fillets from Sea weight_2_ (Kause et al., 2007b; Table 1).

### Genetic trend analysis

In the genetic trend analysis, the phenotypic and genetic parameters of all 10 traits in Table 1 were first generated, and then breeding values of the traits were estimated to assess the genetic changes in the traits across the generations. Multitrait animal models were used to estimate the genetic parameters and breeding values of the traits.

In step one, the phenotypic and genetic parameters of the traits that are routinely recorded in the program (Table 1) were estimated using a single 7-trait model (Table 2). The parameters are presented in Supplementary Appendix 1. The variance components were analysed with the data of the year classes 2001–2007 with the following sample sizes: Weight_1_ (200 737), Weight_2_ (36 937), Weight_3_ (30 744), Sea weight_2_ (33 481), Sea gutted weight_2_ (29 709), Sea visceral% (29 694), and Sea survival_2_ (49 964). The genetic parameters of these traits have been published and discussed previously (Kause et al., 2003, 2005; 2007b; Vehviläinen et al., 2012). Phenotypic and genetic (co)variances were estimated using multitrait linear animal models and DMU software applying the restricted maximum likelihood (REML) method (Madsen and Jensen, 2013). Survival was analyzed on the observed binary scale using a linear mixed model.

**Table 2.** Statistical models^1^ for multitrait animal models used to estimate phenotypic and genetic parameters and breeding values

Trait	Random effects		Fixed effects					Fixed covariate
	Anim	Year×Tank	Year	Year× Stat	Year×Stat× Sex×Mat	Year×Stat× Catar	Year×Stat× Defor	Tsum(year)
Weight_1_	x	x	x					x
Weight_2_	x	x			x	x	x	
Weight_3_	x	x			x	x	x	
Sea weight_2_	x	x			x		x	
Sea gutted weight_2_	x	x			x		x	
Sea visceral%	x	x			x		x	
Sea survival_2_	x	x		x				
Sea FCR_Ind_	x	x	x					
Sea fillet%	x	x	x					
Sea muscle lipid%_BW_	x	x	x					

Model terms are Anim, genetic effect of an individual with full pedigree; Year×Tank, random interaction of birth year and family rearing tank; Year, fixed effect of birth year; Year×Stat, fixed interaction of birth year and testing stations in fresh and sea water; Year×Stat×Sex×Mat, fixed interaction of birth year, station, sex, and maturity; Year×Stat×Catar, fixed interaction of birth year, station, and cataract score; Year×Stat×Defor, fixed interaction of birth year, station, and deformity class; Tsum(year), fixed covariate of cumulative temperature sum at date of recording, nested within birth year.

In step two, the phenotypic and genetic (co)variances of traits Sea FCR_Ind_, Sea fillet% and Sea muscle lipid%_BW_ were merged into the (co)matrices estimated in step one. These three traits were recorded only in one or two generations to estimate their phenotypic and genetic parameters (Kause et al., 2007b, 2016). It was assumed that Sea FCR_Ind_ had a mean of 1.26, which was the mean in 2002 (the year when the fish were evaluated for the FCR) in the farm-level data of FCR_Farm_, and the variances of Sea FCR_Ind_ were scaled to this mean value. This put the estimated genetic trend of Sea FCR_Ind_ at the same scale as the farm-level data on FCR_Farm_. The merging of the three traits resulted in full 10-trait (co)variance matrices. The only unpublished correlation was the one between Sea FCR_Ind_ and Sea fillet%. This needed to be estimated, and the dataset of Kause et al. (2008, 2016) in which both traits are present was used. In these data, fillet% was calculated as the area of the fillet from the total area of the fish body, determined using image analysis of a cross section of a fish (Kause et al., 2008). To be able to use the additive genetic (co)variance matrix of the 10 traits in step four to estimate EBVs, the matrix was bent to be positive definite using the method of Hayes and Hill (1981) (Supplementary Appendix 1).

In step three, the full phenotypic data of the traits routinely recorded in the breeding programme (Table 1) were updated with dummy observations for the traits Sea FCR_Ind_, Sea fillet% and Sea muscle lipid%_BW_. For all fish in the data, these three traits were added to the data with a missing value for all individuals. This allowed us to estimate their genetic trend because their genetic (co)variance with all the other traits was included in the breeding value estimation.

In step four, the updated full phenotypic data, the phenotypic and genetic parameters, the full pedigree, and the statistical models of Table 2 were used to estimate breeding values across all the generations for the 10 traits using MiX99 software to solve mixed model equations (Lidauer et al., 2017). This allows one to partition changes in a phenotypic mean of a trait across generations into its components and genetic and environmental trends. The genetic changes occurring in Sea FCR_Ind_, Sea fillet% and Sea muscle lipid%_BW_ were correlated genetic changes because these traits were not directly selected.

The random and fixed factors and the covariates used in the statistical models to estimate (co)variances and EBVs are presented in Table 2. The full pedigree and all relationships between all animals were accounted for in the analysis to estimate genetic effects. The random Year×Tank effect was used to estimate the common environmental variance of full sibs, including the family tank effect. Residual covariance was always set to zero when estimating EBVs and when calculating genetic correlations between traits that had no records from the same individuals. Phenotypic variance was estimated as *V*_P_ = *V*_G_ + *V*_C_ + *V*_R_, where *V*_G_ is additive genetic variance, *V*_C_ is nongenetic variance in common full sibs, and *V*_R_ is residual variance. Heritability was calculated as *h*^2^ = *V*_A_/*V*_P_, and the common environment ratio was calculated as *c*^2^ = *V*_C_/*V*_P_.

In the final step, the genetic trends for each trait were obtained by calculating the average EBV for each year class of the nucleus fish and plotting the averages against the birth year of the fish. The year 1992 is the first year class with phenotypes and was used as the base in which all EBVs were zero.

A farmer rearing genetically improved fish will benefit from improvements in both Sea FCR_Ind_ and Sea Survival_2_ because both influence the farm-level FCR_Farm_.

Unlike individual-level FCR_Ind_, farm-level FCR_Farm_ is influenced by both the FCR and the potential mismatch between feeding and the real fish biomass in a net pen. Mortality causes a change in fish biomass that is not always observed at a farm. To assess the maximum effect of this combined effect, a hypothetical scenario was imagined in which it was assumed that a farmer is unable to adjust the amount of feed provided to fish biomass in a net pen. Hence, with the survival of genetically improved fish, a farmer will obtain more fish biomass with the same amount of feed provided. To reflect this scenario, a new EBV was calculated (EBV-FCR_Ind+Surv_) that combined the effect of EBV-FCR_Ind_ and EBV-Sea survival_2_. In other words, EBV-FCR_Ind+Surv_ = EBV-FCR_Ind_ (1+Sea survival_2_), meaning that the fish biomass reared is increased proportionally to improved EBV-Sea survival_2_.

The genetic trends of EBV-FCR_Ind_ and EBV-FCR_Ind+Surv_ were integrated into the annual trend of farm-level FCR_Farm_ by using 1993 as the first year when fish from the breeding programme were at sea (i.e., year class 1992 fish at on-growing at the age of 1–2 yr). FCR_Farm_ was set to zero for that year, and its improvement across years was quantified as the deviation from zero. Thereafter, the rescaled FCR_Farm_ and the genetic trends were plotted onto the same graph.

### Economic benefit of using genetically improved fish material

**Figure 1.**
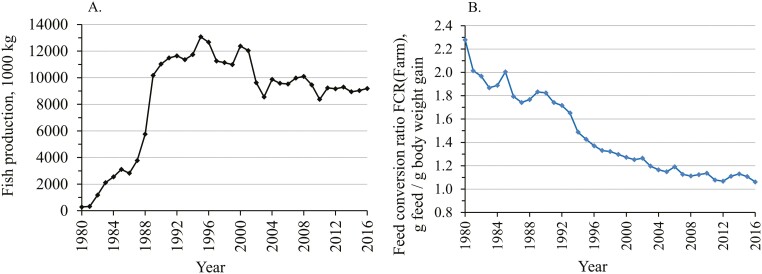
Trend of production volume (A) and farm-level feed conversion ratio, FCR_Farm_ (B), at commercial fish farms located at the coastal areas of the mainland Finland during 1980–2016.

The economic benefit of using the fish material that has been genetically improved for FCR_Ind_ was approximated by calculating current-day farm performance when either the genetically improved rainbow trout of 2016 were assumed to be used in on-growing or when the base population of fish from 1992 were farmed. The following assumptions were made. A total of 9.2 M kg is farmed. This was the volume of farmed fish in year 2016 (Figure 1). Based on the economic analysis of the costs and returns of rainbow trout farming, the feed cost was 1.382 €/kg of feed, and all other costs of rearing until fish are harvested and gutted were 3.22 €/kg of ungutted fish (Kankainen et al., 2016a). A farm sells ungutted fish, and the gutting yield (100 gutted weight/ungutted body weight) was assumed to be 82.9%. The producer price that a farmer obtained when selling the fish was 4.83€/kg of gutted fish.

**Figure 2.**
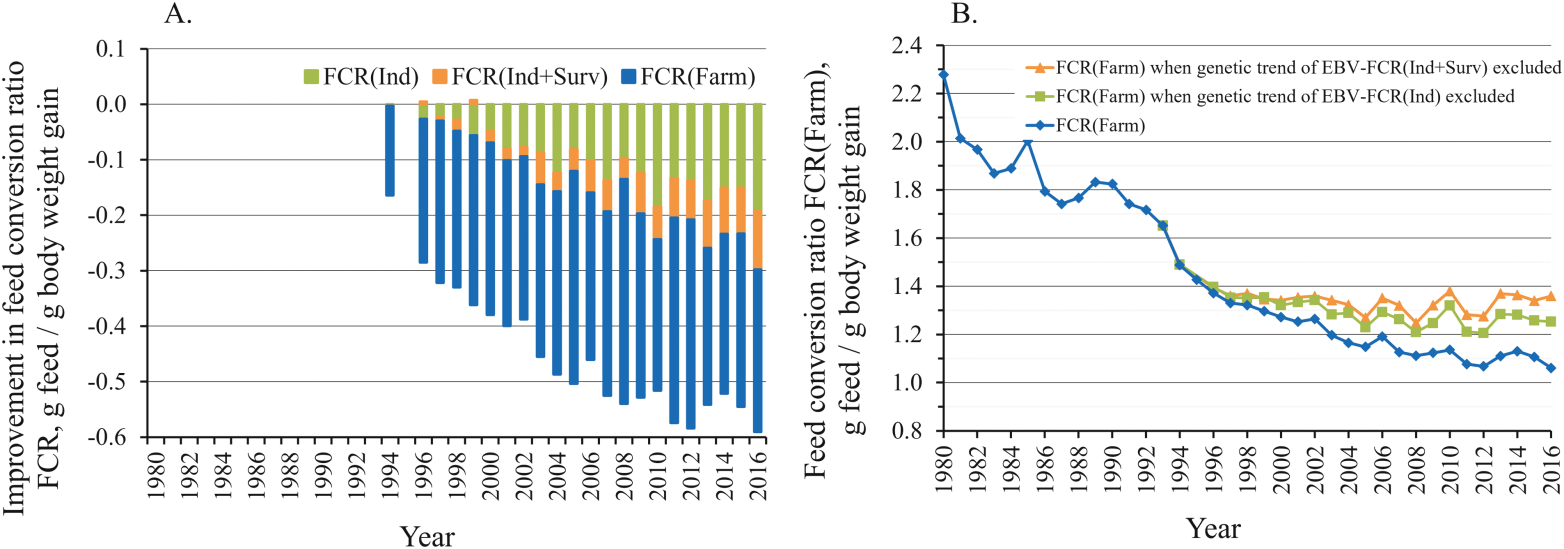
(A) Trend in genetic improvement of EBV-FCR_Ind_ and EBV-FCR_Ind+Surv_ that accounts also for improvement in survival, in relation to phenotypic improvement in farm-level FCR_Farm_. (B) Trend of the observed farm-level FCR_Farm_, and re-calculated FCR_Farm_ when the genetic trend of EBV-FCR_Ind_ or EBV-FCR_Ind+Surv_ has been subtracted from FCR_Farm_.

Then, two scenarios were calculated: one with the observed FCR_Farm_ value of 1.061 from 2016 (scenario: genetically improved fish; Figure 1) and the other scenario with an FCR_Farm_ of 1.253 that excludes genetic improvement (1.061 + 0.192 of genetic improvement from Figure 2) (scenario: base population of fish). The reduction in feed and production costs was calculated. When the producer price is maintained fixed in both scenarios, the reduced feed costs are directly transformed to profit.

## Results

### The scale of aquaculture operations

The data from 1980 to 2016 on biomass rearing and FCR_Farm_ were obtained from all the licenced fish farms located along the mainland coast of Finland (Figure 1). The scale of aquaculture activity, measured as biomass growth, increased sharply from 1980 to the early 1990s. Thereafter, the volumes decreased and stabilized. In the early 1990s, production was approximately 12 million kg, and in 2016, it was 9.2 million kg (Figure 1A). The mainland coast accounts for over half of the total production in Finland, and the other main production area is the Åland Islands.

### Improvement in the FCR

The farm data showed that from the peak year of 1980 to 2016, the FCR_Farm_ recorded at farms improved by 53.4% (Figure 1B). In 1980, the FCR_Farm_ was 2.28, and in 2016, it was 1.06. An FCR value of 1.06 means that to produce 1 kg of fish, 1.06 kg of feed is needed. Hence, to produce 1 kg of fish today, only half of the feed is needed now compared to the 1980s.

Since 1993, when the FCR_Farm_ was 1.65, it has improved by 35.8%, that is, −0.590 FCR units (Figure 1B). Since then, genetically selected rainbow trout have been available for on-growing. The genetic trends of the key individual traits of the breeding programme are in Supplementary Appendix 2. The genetic trend analysis showed that since 1993, the FCR_Ind_ has genetically improved by 1.74% per generation and cumulatively by 11.6%, i.e., by −0.192 FCR units (Supplementary Appendix 2; Figure 2A). This cumulative genetic improvement in the FCR_Ind_ is equal to 32.6% of the total phenotypic improvement in the FCR_Farm_ that occurred at the same time.

Simultaneously, growth at sea, Sea weight_2_, has genetically improved by 57.2%, that is, 6.7% per generation (Supplementary Appendix 2).

The genetic trend for Sea survival_2_ showed a 7.8% percentage point improvement in survival (Supplementary Appendix 2). The genetic trend for EBV-FCR_Ind+Surv_, which includes both the FCR_Ind_ and the potential mismatch between feeding and fish biomass due to mortality, shows that during 2014, 2015, and 2016, the cumulative genetic improvement in EBV-FCR_Ind+Surv_ was equal to 44.9%, 42.9%, and 50.5% of the phenotypic improvement of farm-level FCR_Farm_, respectively (Figure 2A), up from 32.6% if only individual-level FCR_Ind_ was accounted for.

### Reduction in specific phosphorus and nitrogen loading

**Figure 3.**
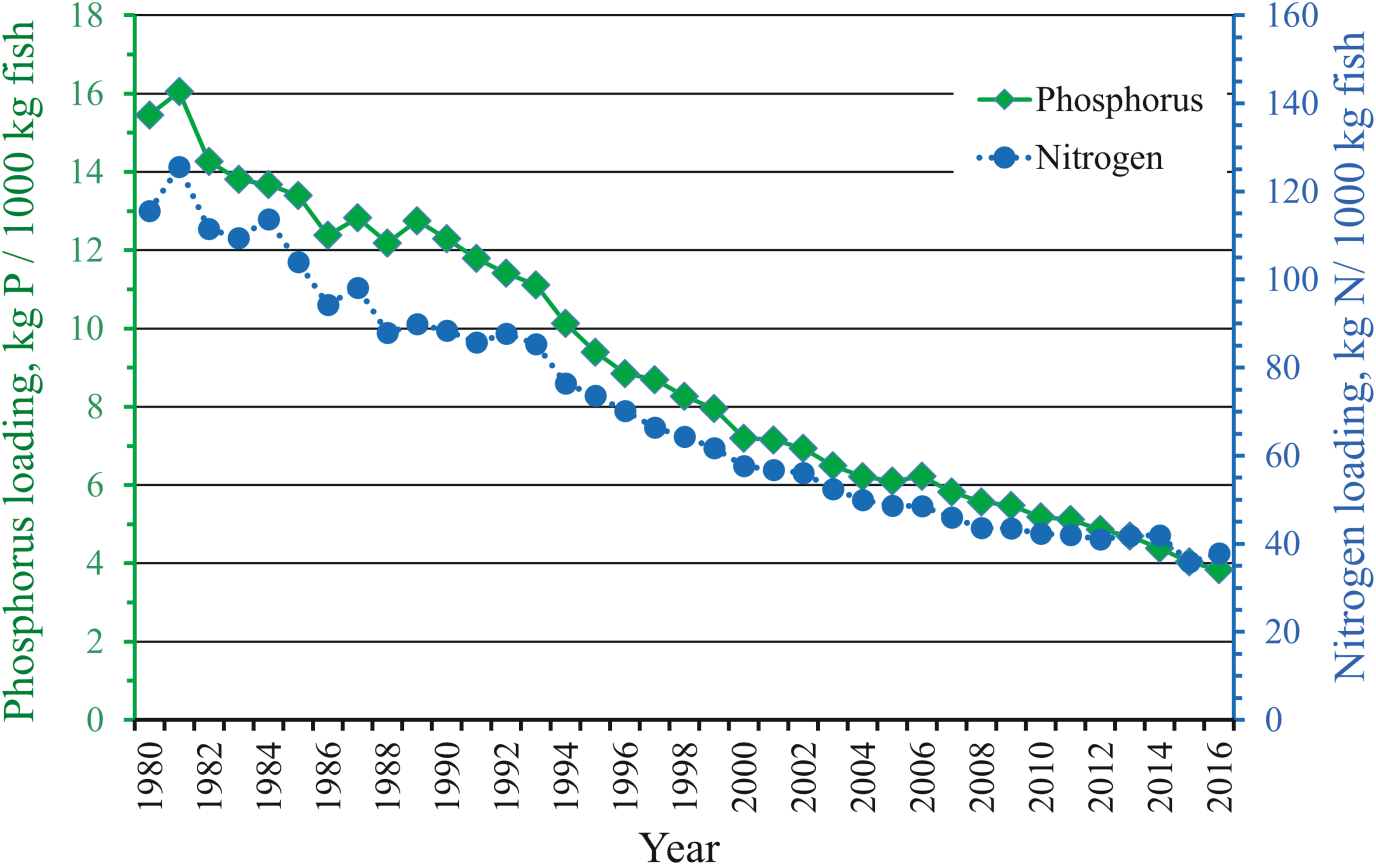
Specific phosphorus and nitrogen loading to water from commercial rainbow trout farms located at the coastal areas of the mainland Finland during 1980–2016.

The farm data showed that from the peak year of 1981, specific nitrogen and phosphorus loading to water from rainbow trout farms was reduced by 76% and 70%, respectively (Figure 3). In 1981, the loading of phosphorus was 16.0, and that of nitrogen was 125.6 (kg/1000 kg of fish). In 2016, the values were 3.8 and 37.8, respectively. The reduction in the FCR_Farm_ was smaller (max 53.4%) than the reduction in specific loadings (>70%), indicating that the latter is not just a consequence of the former but that other factors are also involved.

Assuming that the reduction in specific N and P loading is directly related to the genetic improvement in the FCR_Ind_ with a cumulative change of 11.6%, the consequent cumulative genetic reduction in specific N and P loading should also be 11.6%.

### Economic benefit of using genetically improved fish material

Figure 2B shows the level at which farm-level FCR_Farm_ would have been if no selective breeding had been practiced. If there had not been genetic improvement during the last two decades, the phenotypic improvement in the FCR_Farm_ would have flattened. This is also evident in Figure 2A, in which the degree of genetic improvement expands rapidly across the years. In 2016, the FCR_ Farm_ was 1.061 (including genetic improvement), and the FCR_Farm_ would have been 1.253 without genetic improvement in EBV-FCR_Ind_ (−0.192), a difference of 18.1%.

Under present-day conditions of growing, the use of genetically improved fish for the FCR_Ind_ reduces feed costs by 18.1% and total production costs by 7.8% at commercial farms (Table 3). When 9.2 M kg of fish are farmed, the annual total feed costs are reduced by −2 443 060€. The reduction in feed costs is −0.266€ and −0.321€ per kg of ungutted and gutted fish, respectively.

**Table 3.** Expected economic impact of using either (a) base-population fish or (b) selected fish with improved FCR_Ind_^1^ on farm costs and returns

Cost, return, profit^2^	(a) Base-population fish	(b) Genetically improved fish	Change	% improvement
**A.** Total production, kg ungutted fish	9,188,000	9,188,000		
**B.** Feed cost per kg feed, €/kg feed	1.382	1.382		
**C.** Rearing cost per kg fish, €/kg ungutted fish	1.952	1.952		
**D.** Total rearing costs, € (**A×C)**	17,934,976	17,934,976		
**E.** FCR_Ind_, kg feed delivered/ kg fish biomass gain	1.253	1.061	−0.192^1^	18.1%
**F.** Total feed needed, kg (**A×E**)	11,516,239	9,748,468	−1,767,771	18.1%
**G.** Total feed cost, €) (**F×B**)	15,915,443	13,472,383	−2,443,060	18.1%
**H.** Feed cost per kg fish, €/kg ungutted fish (**G/A**)	1.732	1.466	−0.266	18.1%
I. Total production costs, € (**D+G**)	33,850,419	31,407,359	−2,443,060	7.8%
**J.** Total production cost per kg fish, €/kg ungutted fish (**I/A**)	3.684	3.418	−0.266	7.8%
**K.** Total production of gutted fish, kg gutted fish (**A×0.829 yield**)	7,616,852	7,616,852		
**L. Return:** Producer price per kg gutted fish, €/kg gutted fish^3^	4.830	4.830		
**M. Cost:** Total production cost of kg gutted fish, €/kg gutted fish(**I/K**)	4.444	4.123	−0.321	7.8%
**N. Profit:** Profit per kg gutted fish, €/kg gutted fish (**L-M)**^4^	0.386	0.707	0.321	118%

Genetic improvement estimated in FCR_Ind_ in Figure 2.

Capital letters indicate the formulas used to calculate the values.

Price that a farmer gets from fish when selling it.

Assumes that the reduced feed costs can be directly transformed to be profit, as the producer price is maintained fixed.

If the producer price remains fixed in the two scenarios and the cost reduction (0.321 €/kg of gutted fish) is directly translated on the top of the original profit (0.272 €/kg of gutted fish), the profit increases by 118%.

## Discussion

Resource efficiency, the ratio of inputs to outputs, is essential for both the economic and environmental performance of any sector of food production. The data from commercial rainbow trout farms located along the coast of mainland Finland show that the farm-level feed conversion ratio (FCR_Farm_) has improved from the 1980s onwards by 53.4%, and specific nitrogen and phosphorus loadings have decreased by over 70%. Feed costs are ~43% of the total costs of primary fish production in Finland (Kankainen et al., 2016a, b), and hence, an improved FCR leads to a major improvement in the cost structure of aquaculture.

These improvements in resource efficiency are a win–win for both industry and the environment—the same amount of seafood can be produced with significantly reduced amounts of raw materials and reduced environmental impact. In Finland, licensing of fish farms and production volumes that can be farmed are based on the environmental assessment in which nutrient loading, especially phosphorus and the ecological state of the aquatic environment, is key parameters. In 2008–2012, aquaculture accounted for 2.3% of phosphorus loading from Finland to the Baltic Sea (SYKE, 2015). The majority originates from agriculture, forestry, and human communities. The reduction in the environmental impact is one key factor for the development of more sustainable aquaculture production. Our results show that in Finland, the nutrient loading of aquaculture has been dramatically reduced during the last decades.

### Factors explaining improvement in the FCR

At least three factors explain the decades-long improvement in the feed conversion ratio as well as N and P loading. First, it is essential to realize that the first data points on farm performance originate from the early 1980s, when fish farming was in its early stages in Finland. Feeding practices and farm management have improved ever since, and the current farms are larger and more efficient. In particular, fish are not overfed; overfeeding leads to uneaten feed and a high FCR. Overfeeding can be avoided by adjusting feeding to match the known fish biomass, typically summarized in feeding tables, and by observing the prevalent appetite, feeding activity, biomass, and satiation level of the fish in a net pen.

Second, feed composition has been changed by the feed industry to better match the nutritional needs of fish, and the digestibility of feed ingredients has been improved by processing and feed additives (Halver and Hardy, 2002; Hardy, 2010; NRC, 2011; Ytrestøyl, et al. 2015). This reduces the surplus of feed waste. However, not all efforts have improved the FCR. For instance, the inclusion of plant-based raw materials into the feed of carnivorous fish may the FCR, especially in the early stages of their inclusion when their processing was not yet advanced (Hardy, 2010; Ytrestøyl et al., 2015).

Regarding phosphorus, the main mechanisms to reduce effluent from feed have been to reduce phosphorus content in the feed and to increase its digestibility. This has been achieved, for example, by including phytase enzymes into feeds that consist of plant-based raw materials. Phytate is the main storage form of phosphorus in many plants, but phytate-bound phosphorus is not digestible to most fishes. Phytate enzymes, which can be added to feed, convert phytate into a form that can be utilized by fish. Experiments with phytate enzymes have documented decreases in the total phosphorus load to the environment from 30% to 100% in species such as tilapia, salmonids, carp, channel catfish, and shrimp (Cao et al., 2007; Lemos and Tacon, 2017). In Finland, phytase enzymes were introduced into commercial fish feed in 2009 with an expected decrease of 26% in phosphorus loading (Rehuraisio Oy, 25.3.2019, press release). The need to reduce the amount of phosphorus in the feed is counterbalanced by the physiological needs of fish because phosphorus is an essential nutrient for the growth, skeletal development and reproduction of fish.

### Genetic improvement

The third factor explaining the improvement in the FCR, the special focus in the current study, is selective breeding.

Genetic improvement in the FCR_Ind_ was 1.74% per generation and 11.6% cumulatively across seven episodes of selection across generations. The national breeding program was established in 1989–1990 by crossing four strains, and the first families were generated in 1992 (Siitonen, 1986; Kause et al., 2005). The use of modern fish material genetically improved for the FCR, instead of using the base population of fish, reduces feed costs by 18.3%, that is, 0.266€ per kg of ungutted fish.

Before the breeding program was started, broodstock companies in Finland had been practicing mass selection for fast growth in freshwater. This is expected to improve the FCR at commercial sea farms but less efficiently than the national breeding program. Regarding growth, the genetic correlation between fresh and seawater production environments, which quantifies genotype reranking across the environments (GxE), is only 0.65 (Supplementary Appendix 1; Kause et al., 2005). In addition, the phenotypic correlation between growth and the FCR is typically modest at −0.34, whereas the genetic correlation is much stronger (Kause et al., 2016). For mass selection of growth, it is the phenotypic correlation that determines the strength of co-selection on FCR, as no genetic information is utilized in mass selection. These two factors reduce the power of mass selection in freshwater to improve the FCR at sea. The national breeding program that tests families in both fresh and seawater farms in a split-family design accounts for GxE and selects animals with the best EBVs for the traits recorded at sea, not just the best phenotypes recorded in fresh water (Kause et al., 2005). The selection criteria of the national breeding program include growth as well as traits that are genetically correlated with the FCR (Kause et al., 2016; Knap and Kause, 2018; Supplementary Appendix 1), making the improvement of the FCR more efficient.

Our results showed that survival under commercial conditions was genetically improved by 7.8% percentage points across generations. A hypothetical scenario in which it was assumed that feeding was not adjusted for fish biomass loss due to mortality revealed that genetic improvement would have contributed to 44.9%–50.5% of the phenotypic improvement in the FCR_Farm_, up from 32.6% if only individual-level FCR_Ind_ was accounted for. It is good to note that the farm-level FCR_Farm_ is calculated based on the feed provided at the farm and the harvested fish biomass. Farm-level FCR_Farm_ may be influenced by mortality, and the farms that assess biomass in production and adjust feeding accordingly improve their FCR_Farm_. This is the norm today. However, during the early decades of farming, the mismatch between feeding and fish biomass was expected to have been large. The results highlight that mortality that can be influenced by breeding is a major determinant of the resource efficiency of a farm if mortality is not accounted for in feeding.

For phosphorus and nitrogen loading, a simple assumption can be made that compared to the use of the base population, modern fish with a genetically improved FCR reduce N and P flow into water from farms by 18.3%. This assumes that 18.3% more of the feed with N and P is retained as fish biomass and its N and P reserves. This is a feasible scenario when focusing only on the improvement in the FCR. This does not reflect all the potential impacts that selective breeding can have on nutrient loading. The amount of N and P loading is also impacted by changes in body composition and retention efficiencies of N and P. More detailed analyses are needed to quantify these impacts. If N and P retention of fish is improved by a selection program, as the initial evidence suggests for our rainbow trout (Kause et al., 2016), the present estimates for the reduction in specific N and P loading are too low. Retention efficiencies of N and P would be key traits to be improved by selection (and feed development), but their recording on individual fish is very difficult. There are a limited number of genetic studies on retention efficiencies, and the results are contradictory and with variable methodology (Thodesen et al., 1999; Kause et al., 2016; Gjedrem and Rye, 2018; Dvergedal et al., 2019).

### Direct vs. indirect selection

The most effective way to improve the FCR by selection would be to select directly for the FCR (Kause et al., 2006, 2016). This is not done in any fish breeding program because this would require recording of feed intake on thousands of individual fish. This is a major challenge regarding fish. To overcome this issue, indirect selection via growth and body composition has been advocated as an effective alternative to improve the FCR (Quinton et al., 2007, Quillet et al., 2007; Kause et al., 2016; Knap and Kause, 2018). In the Finnish national breeding program for rainbow trout, multiple traits are selected and improved that are expected to improve the FCR indirectly, namely, growth, Sea muscle lipid_BW_%, Sea visceral%, and Sea Fillet%.

In farmed fish, selection for growth is expected to improve the FCR only moderately or not at all. This is a simple consequence of the genetic correlation between the FCR and growth ranging between 0.0 and −0.6, depending on the fish species (Knap and Kause, 2018). In fact, in some species such as brown trout (*Salmo trutta* L.), it has been shown that improvement of growth by selection does not improve the FCR (Mambrini et al., 2004). This is most likely because feed intake rather than feed utilization is the first trait to respond to selection for faster growth in species with limited or no domestication history.

Both fillet% and lipid deposition are genetically related to the FCR in rainbow trout (Supplementary Appendix 1; Quillet et al., 2007; Kause et al., 2016). Lean, high fillet% fish are expected to have a favorable FCR due to the lower energy needed to deposit wet weight growth compared to fatty fish (Jobling, 1994; Kause et al., 2016; Knap and Kause, 2018). In the selection index of the Finnish breeding program for rainbow trout, Sea visceral% (% of viscera of wet body weight) is included in the selection index and selected against. This is to reduce excessive lipid deposition in the body cavity, as well as to improve Sea fillet%, given that the genetic correlation between Sea visceral% and Sea fillet% is highly negative, −0.71, and Sea visceral% has a higher heritability of 0.58 compared to 0.29 of Sea fillet% (Kause et al., 2007b; Supplementary Appendix 1). The FCR_Ind_ has a genetic correlation of −0.50 with Sea fillet% (which is genetically increased by selection), +0.54 with Sea muscle lipid_BW_% (which is genetically decreased by selection), and +0.10 with Sea visceral% (which is genetically decreased) (Supplementary Appendix 1; Kause et al., 2016). Genetic changes in these traits are hence likely to improve the FCR_Ind_.

In our study, genetic improvement in growth was 6.7% per generation. In a review of genetic responses in farmed fish, the average genetic gain for growth was 12.7% (Gjedrem and Rye, 2018). Our estimate is at the lower end of the observed range. In our national breeding program, multitrait selection in which the nucleus is not the production environment has been performed with up to 15 traits included in the selection index, and hence selection for growth is not that strong. In the review of Gjedrem and Rye (2018), many examples have only a few generations, and selection has been performed only for growth in a single environment.

Reliable estimates for genetic improvement in the FCR in farmed fish are limited (Gjedrem and Rye, 2018). For instance, Thodesen et al. (1999) reported that Atlantic salmon selectively bred for rapid growth utilized feed better than their wild counterparts. In line with our analysis, Quillet et al. (2007) reported realized genetic improvement in the FCR in the line selected for low muscle lipid%, corrected for body weight, in rainbow trout. de Verdal et al. (2022) observed a 12% genetic difference in tilapia lines that underwent divergent selection for the FCR. The study of Mambrini et al. (2004) on wild brown trout showed no genetic improvement in the FCR in response to major genetic changes in growth rate. It has been suggested that growth selection may also lead to more aggressive fish, rather than preferring the fish with the highest genetic potential for growth and the FCR, but the evidence for this is not conclusive (Doyle and Talbot, 1986; Janhunen et al., 2012; de Verdal et al., 2019).

## Conclusions

To conclude, industry-level farm data combined with the multigenerational analysis of a rainbow trout breeding program imply that the feed conversion ratio at commercial farms has improved by over 53% and that the specific nutrient loading has been reduced by up to 70%. The genetic progress obtained with the national breeding program is expected to be over 30% of this improvement. These improvements have major positive effects on the cost structure of farms and on their environmental footprint.

## Supplementary Data

Supplementary data are available at *Journal of Animal Science* online.

skac214_suppl_Supplementary_Appendix_S1

skac214_suppl_Supplementary_Appendix_S2

skac214_suppl_Supplementary_Appendix_S2_Legend

## Abbreviations

EBV: estimated breeding value
EBV-FCR_Ind+Surv_: EBV of the feed conversion ratio that accounts for the FCR and fish survival
FCR: feed conversion ratio
FCR_Farm_: farm-level feed conversion ratio
FCR_Ind_: individual-level feed conversion ratio
Sea weight_2_: body weight at sea after two growing seasons
Sea survival_2_: survival at sea from tagging until the end of one growing season in the sea
Sea gutted weight_2_: gutted body weight at sea after two growing seasons
Sea visceral%: percentage of entrails out of body weight at sea after two growing seasons
Sea FCR_Ind_: feed conversion ratio recorded for individuals at sea
Sea muscle lipid%_BW_: muscle lipid percentage at sea, statistically corrected for body weight
Sea fillet%: percentage of fillet weights out of body weight at sea
Weight_1_: body weight at tagging after one growing season in fresh water
Weight_2_: body weight after two growing seasons in fresh water
Weight_3_: body weight after three growing seasons in fresh water
