# Supplementary material for: Improvement in feed efficiency and reduction in nutrient loading from rainbow trout farms: the role of selective breeding
Source: J Anim Sci. 2022 Jun 9;100(8):skac214. doi: 10.1093/jas/skac214 (PMC9387595; doi:10.1093/jas/skac214)
Supplement: skac214_suppl_Supplementary_Appendix_S1 [file skac214_suppl_supplementary_appendix_s1.docx]

**Appendix 1A.** Genetic correlations (below diagonal) and heritability estimates (*h*^2^ on diagonal) of the traits used to estimate breeding values.

|  | Weight_1_ | Weight_2_ | Weight_3_ | Sea weight_2_ | Sea gutted weight_2_ | Sea visceral% | Sea survival_2_ | Sea FCR_Ind_ | Sea fillet% | Sea muscle lipid%_BW_ |
| --- | --- | --- | --- | --- | --- | --- | --- | --- | --- | --- |
| Weight_1_ | **0.26** |  |  |  |  |  |  |  |  |  |
| Weight_2_ | 0.38 | **0.33** |  |  |  |  |  |  |  |  |
| Weight_3_ | 0.17 | 0.79 | **0.32** |  |  |  |  |  |  |  |
| Sea weight_2_ | 0.27 | 0.65 | 0.44 | **0.32** |  |  |  |  |  |  |
| Sea gutted weight_2_ | 0.29 | 0.64 | 0.43 | 0.99 | **0.32** |  |  |  |  |  |
| Sea visceral% | -0.16 | 0.11 | 0.10 | 0.10 | -0.01 | **0.58** |  |  |  |  |
| Sea survival_2_ | -0.04 | 0.01 | -0.00 | 0.45 | 0.46 | -0.09 | **0.05** |  |  |  |
| Sea FCR_Ind_ | -0.05 | -0.25 | -0.20 | -0.47 | -0.50 | 0.11 | 0.00 | **0.07** |  |  |
| Sea fillet% | 0.00 | 0.02 | 0.01 | 0.04 | 0.14 | -0.71 | 0.00 | -0.50 | **0.29** |  |
| Sea muscle lipid%_BW_ | -0.05 | -0.04 | -0.15 | -0.28 | -0.25 | 0.05 | 0.00 | 0.54 | 0.12 | **0.42** |

Standard errors of the correlations range between 0.0006 and 0.088.

**Appendix 1B.** Common-environment correlations (below diagonal) and proportion of variance explained by common environmental effects (*c*^2^ on diagonal) of the traits used to estimate breeding values.

|  | Weight_1_ | Weight_2_ | Weight_3_ | Sea weight_2_ | Sea gutted weight_2_ | Sea visceral% | Sea survival_2_ | Sea FCR_Ind_ | Sea fillet% | Sea muscle lipid%_BW_ |
| --- | --- | --- | --- | --- | --- | --- | --- | --- | --- | --- |
| Weight_1_ | **0.28** |  |  |  |  |  |  |  |  |  |
| Weight_2_ | 0.44 | **0.08** |  |  |  |  |  |  |  |  |
| Weight_3_ | 0.27 | 0.67 | **0.06** |  |  |  |  |  |  |  |
| Sea weight_2_ | 0.72 | 0.56 | 0.52 | **0.05** |  |  |  |  |  |  |
| Sea gutted weight_2_ | 0.72 | 0.55 | 0.53 | 0.99 | **0.05** |  |  |  |  |  |
| Sea visceral% | -0.09 | 0.17 | -0.03 | 0.12 | 0.08 | **0.01** |  |  |  |  |
| Sea survival_2_ | 0.07 | 0.25 | 0.23 | 0.26 | 0.26 | 0.10 | **0.03** |  |  |  |
| Sea FCR_Ind_^1^ | 0 | 0 | 0 | 0 | 0 | 0 | 0 | **0.05** |  |  |
| Sea fillet%^1^ | 0 | 0 | 0 | 0 | 0 | 0 | 0 | 0 | **0.01** |  |
| Sea muscle lipid%_BW_^1^ | 0 | 0 | 0 | 0 | 0 | 0 | 0 | 0 | 0 | **0.01** |

^1^ - Genetic trends for these traits are estimated only based on their genetic correlations with the traits that are routinely recorded, and hence common-environment correlations are set to zero.

**Appendix 1C.** Residual correlations (below diagonal) of the traits used to estimate breeding values.

|  | Weight_1_ | Weight_2_ | Weight_3_ | Sea weight_2_ | Sea gutted weight_2_ | Sea visceral% | Sea survival_2_ | Sea FCR_Ind_ | Sea fillet% | Sea muscle lipid%_BW_ |
| --- | --- | --- | --- | --- | --- | --- | --- | --- | --- | --- |
| Weight_1_ | **-** |  |  |  |  |  |  |  |  |  |
| Weight_2_ | 0.40 | **-** |  |  |  |  |  |  |  |  |
| Weight_3_ | 0.24 | 0.66 | **-** |  |  |  |  |  |  |  |
| Sea weight_2_ | 0.42 | na | na | **-** |  |  |  |  |  |  |
| Sea gutted weight_2_ | 0.42 | na | na | 0.99 | **-** |  |  |  |  |  |
| Sea visceral% | 0.10 | na | na | 0.18 | 0.12 | **-** |  |  |  |  |
| Sea survival_2_ | 0.06 | na | na | na | na | na | **-** |  |  |  |
| Sea FCR_Ind_ ^1^ | 0 | 0 | 0 | 0 | 0 | 0 | 0 | **-** |  |  |
| Sea fillet% ^1^ | 0 | 0 | 0 | 0 | 0 | 0 | 0 | 0 | **-** |  |
| Sea muscle lipid%_BW_ ^1^ | 0 | 0 | 0 | 0 | 0 | 0 | 0 | 0 | 0 | **-** |

na - Traits are recorded in different environments and hence residual correlations does not exists.

^1^ - Genetic trends for these traits were estimated only based on their genetic correlations with the traits that are routinely recorded, and hence residual correlations were set to zero.
