# Supplementary figures and images for: Improvement in feed efficiency and reduction in nutrient loading from rainbow trout farms: the role of selective breeding
Source: J Anim Sci. 2022 Jun 9;100(8):skac214. doi: 10.1093/jas/skac214 (PMC9387595; doi:10.1093/jas/skac214)

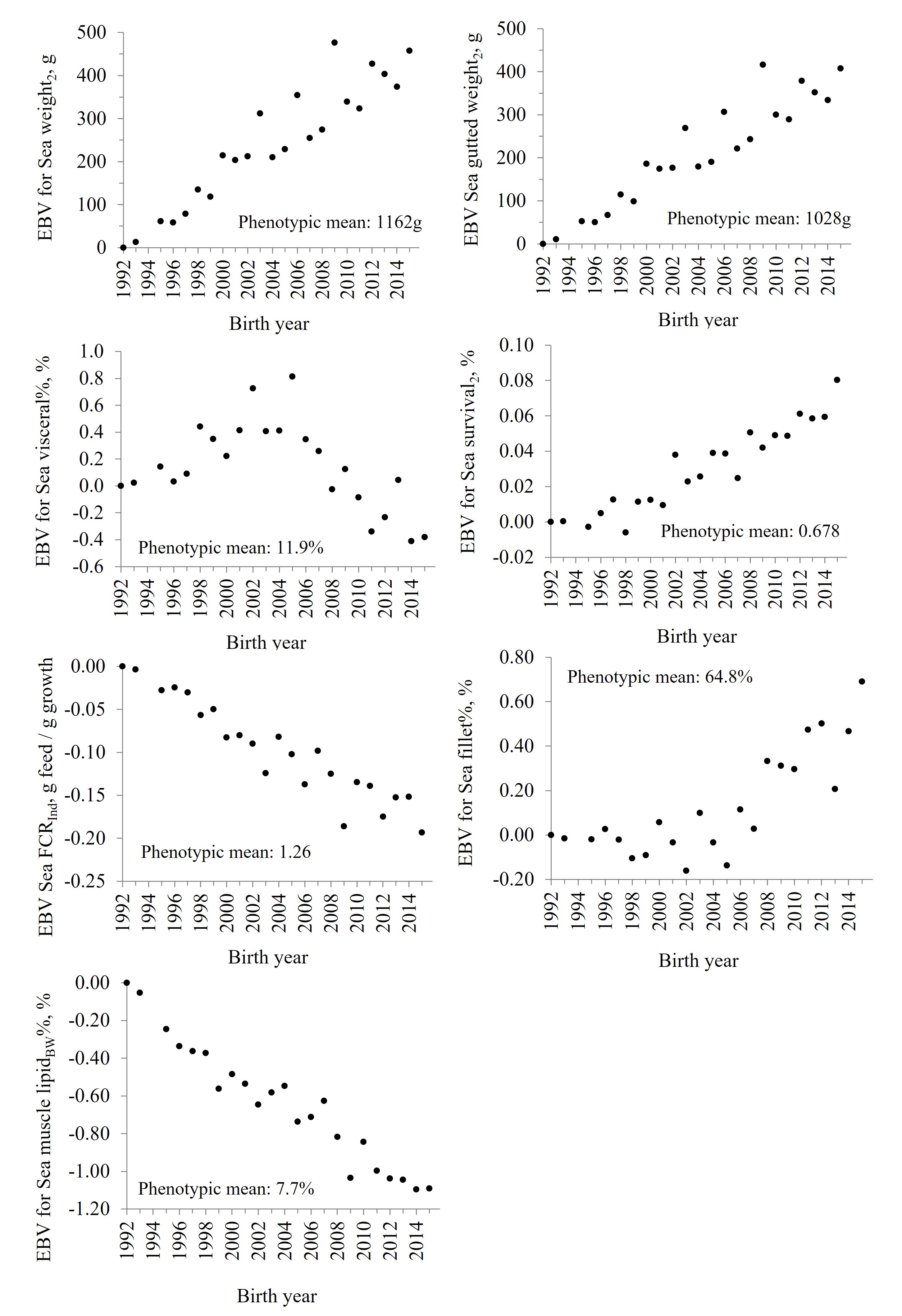

Supplement: skac214_suppl_Supplementary_Appendix_S2 [file skac214_suppl_supplementary_appendix_s2.jpeg]
