# Supplementary material for: Improvement in feed efficiency and reduction in nutrient loading from rainbow trout farms: the role of selective breeding
Source: J Anim Sci. 2022 Jun 9;100(8):skac214. doi: 10.1093/jas/skac214 (PMC9387595; doi:10.1093/jas/skac214)
Supplement: skac214_suppl_Supplementary_Appendix_S2_Legend [file skac214_suppl_supplementary_appendix_s2_legend.docx]

**Appendix 2.** Genetic trends across year classes, given as average estimated breeding values (EBV), for seven key traits for commercial sea farming.
